# Geographical patterns in climate and agricultural technology drive soybean productivity in Brazil

**DOI:** 10.1371/journal.pone.0191273

**Published:** 2018-01-30

**Authors:** Jordana Moura Caetano, Geiziane Tessarolo, Guilherme de Oliveira, Kelly da Silva e Souza, José Alexandre Felizola Diniz-Filho, João Carlos Nabout

**Affiliations:** 1 Campus de Ciências Exatas e Tecnológicas (CCET), Universidade Estadual de Goiás, Anápolis, Goiás, Brazil; 2 Centro de Ciências Agrárias, Ambientais e Biológicas, Setor de Biologia, Universidade Federal do Recôncavo da Bahia (UFRB), Cruz das Almas, Bahia, Brazil; 3 Departamento de Ecologia, Instituto de Ciências Biológicas, Universidade Federal de Goiás, Campus Samambaia, Goiânia, Goiás, Brazil; Universita degli Studi di Trento, ITALY

## Abstract

The impacts of global climate change have been a worldwide concern for several research areas, including those dealing with resources essential to human well being, such as agriculture, which directly impact economic activities and food security. Here we evaluate the relative effect of climate (as indicated by the Ecological Niche Model—ENM) and agricultural technology on actual soybean productivity in Brazilian municipalities and estimate the future geographic distribution of soybeans using a novel statistical approach allowing the evaluation of partial coefficients in a non-stationary (Geographically Weighted Regression; GWR) model. We found that technology was more important than climate in explaining soybean productivity in Brazil. However, some municipalities are more dependent on environmental suitability (mainly in Southern Brazil). The future environmental suitability for soybean cultivation tends to decrease by up 50% in the central region of Brazil. Meanwhile, southern-most Brazil will have more favourable conditions, with an increase of ca. 25% in environmental suitability. Considering that opening new areas for cultivation can degrade environmental quality, we suggest that, in the face of climate change impacts on soybean cultivation, the Brazilian government and producers must invest in breeding programmes and more general ecosystem-based strategies for adaptation to climate change, including the development of varieties tolerant to climate stress, and strategies to increase productivity and reduce costs (social and environmental).

## Introduction

The impact of the increase in temperature due to climate change has been a worldwide concern for several research areas [1–7]. Among the various potential impacts of climate change, those of special concern are on resources essential to human wellbeing, like water availability and agriculture, with direct impacts on economic activities and food security [8].

Agriculture is among the human activities most vulnerable to climate change [9, 10]. More specifically, the impacts are related to the shortening of the growth and flowering season, along with reduction of the number and size of grains, as well as the total yield [11, 12]. Consequently, in many countries, the agricultural productivity of crops is impacted in some way by the increase in temperature [13, 14], and many models have predicted that the yield of many crops will continue to decrease in future climatic scenarios [15, 16]. Thus, understanding how climate change can affect agriculture has now become a key step in guaranteeing global food security.

The negative impact of climate change on crop yields is expected to be more severe in low latitudes, affecting mainly developing countries [17]. Countries in these areas, which are highly dependent on agricultural activity, such as Brazil, will suffer great economic damage due to the effect of climate change on crops [18,19]. However, the impact of climate change at low latitudes will probably have consequences for food supply at a global scale, as some countries in the low latitudes are important producers of food grains.

Many studies have investigated the impact of global climate change on the biology and productivity of agricultural plant species [10, 14, 17–21]. Moreover, the availability of environmentally suitable areas is another important agricultural factor that can be affected by climate change. Although contemporary agriculture benefits from investments in high-tech plant production, climate is still critical to the productivity and distribution of the species [22]. Thus, investigating the relative effect of climate and technology on agricultural productivity can help in understanding the impact of global climate change. Despite the fact that the impact of climate change on the environmental suitability of a species has been known for a long time, only recently studies have investigated the impact of climate change on the geographic distribution of agricultural plant species [10, 23–25].

On large spatial scales, species density and distribution are mainly constrained by the climatic conditions in which species can survive [26,27]. Considering that species occur in all climatically suitable places, and are absent from all unsuitable places (i.e., species are in equilibrium with the actual climate; [28,29]), changes in climatic variables will change the suitability of the areas for the species, leading to changes in species density and distribution. As environmental suitability and species density have been shown to be positively correlated [30], future climate change can alter the geographical pattern of distribution and the production of many crop species [31–33].

Ecological niche models (ENMs hereafter) have been one of the most widely used statistical and computational tools to evaluate the environmental suitability of species throughout their geographic range and to generate hypotheses about shifts in their distribution [34–36]. These models relate the occurrence of species with environmental variables, generating response curves that can be used to predict environmental suitability in other areas or over time [35]. ENMs have been usefully applied for a variety of ecological purposes, including assessing the impacts of climate change on species’ environmental suitability and distribution [37,38]. Due to the potential of ENMs to provide information about the spatial structure of species’ environmental suitability over different climates and time, these models have been used to model responses in agricultural species in order to evaluate the impacts of climate change on the future yield.

Agricultural species have been more frequently evaluated using more specific and “mechanistic” models (see Nabout et al. [23] and Estes et al. [39], for comparisons). However, because of their generality and simplicity, correlative ENMs can still be quite useful for quick and empirical predictions. Moreover, studies that associate environmental suitability (obtained by ENM) with demographic aspects of the species (e.g., productivity, density, and frequently others) have used correlation or linear regression models, with results varying on the support or not of this relationship (e.g., Nabout et al. [40, 41], Torres et al. [42] Weber et al. [43], Filz et al. [44], Jiménez-Valverde et al. [45]). However, the relationship of species suitability and its yield may present non-stationary geographical patterns, i.e., can systematically vary from one region to another [46]. For example, it is possible that, in areas with similar suitability values, different agricultural practices can lead to differences in yield. Consequently, linear and regression models that assume a constant relationship over space cannot account for this spatial variability. Thus, geographically weighted regression (GWR) may be a more appropriate approach [47] to evaluate the relationship between species suitability and productivity. The GWR is a statistical technique that captures spatial differences in the relationship of variables by allowing the modelling of processes to vary over the study area. This analysis is frequently used in broad-scale ecological studies (e.g., Terribile and Diniz-Filho [48], Eme et al. [49]), and thus its combination with ENM may be useful to evaluate the changes in environmental suitability over the space [46].

In economic and social terms, soybeans (*Glycine max* (L.) Merrill) are a globally important crop. Soybean production is one of the greatest among the oil crops and is a relevant source of income for producers [50]. In addition, soybeans represents a significant source of protein and calories in human nutrition [51].

Soybeans arrived in Brazil via the United States in 1882, and the first soybean crop in Brazil was recorded in 1901 in Rio Grande do Sul state (in the extreme south), where there were suitable conditions to develop and expand soybean cultivation. Thus, it is likely that the climate conditions prevailing in southernmost Brazil are similar to those of the original distribution of soybeans in the southern US [52]. Additionally, apart from favourable environmental conditions, in the mid-1950s, an official programme encouraged national wheat cultivation in the southern region [52]. It also provided an incentive for soybean cultivation, which was regarded as the best summer alternative to succeed the wheat crop planted in the winter [53]. In the 1960s, soybeans were established as an economically important crop in Brazil, and, in the following decade, soybeans became the main crop of Brazilian agribusiness [52, 54]. Currently, Brazil is the second largest worldwide soybean producer, and the largest in Latin America [55], producing 96,228 thousand tons in an area of ~32,000 thousand hectares (56% of the cultivated areas in Brazil) [56]. Thus, considering the importance of soybean agricultural activity for the global economy, and climate change possibly shifting the present agricultural scenario, here, we aim to evaluate the relative effect of climate (as indicated by ENMs) and agricultural technology on actual soybean productivity in Brazilian municipalities. Moreover, we estimate the future geographic distribution of soybeans, coupling ENM with agricultural technology variables and, using spatial analyses, we are able to address complex local and non-stationary patterns (the Geographically Weighted Regression–GWR).

## Materials and methods

### Productivity data and areas of soybean presence

**Fig 1:**
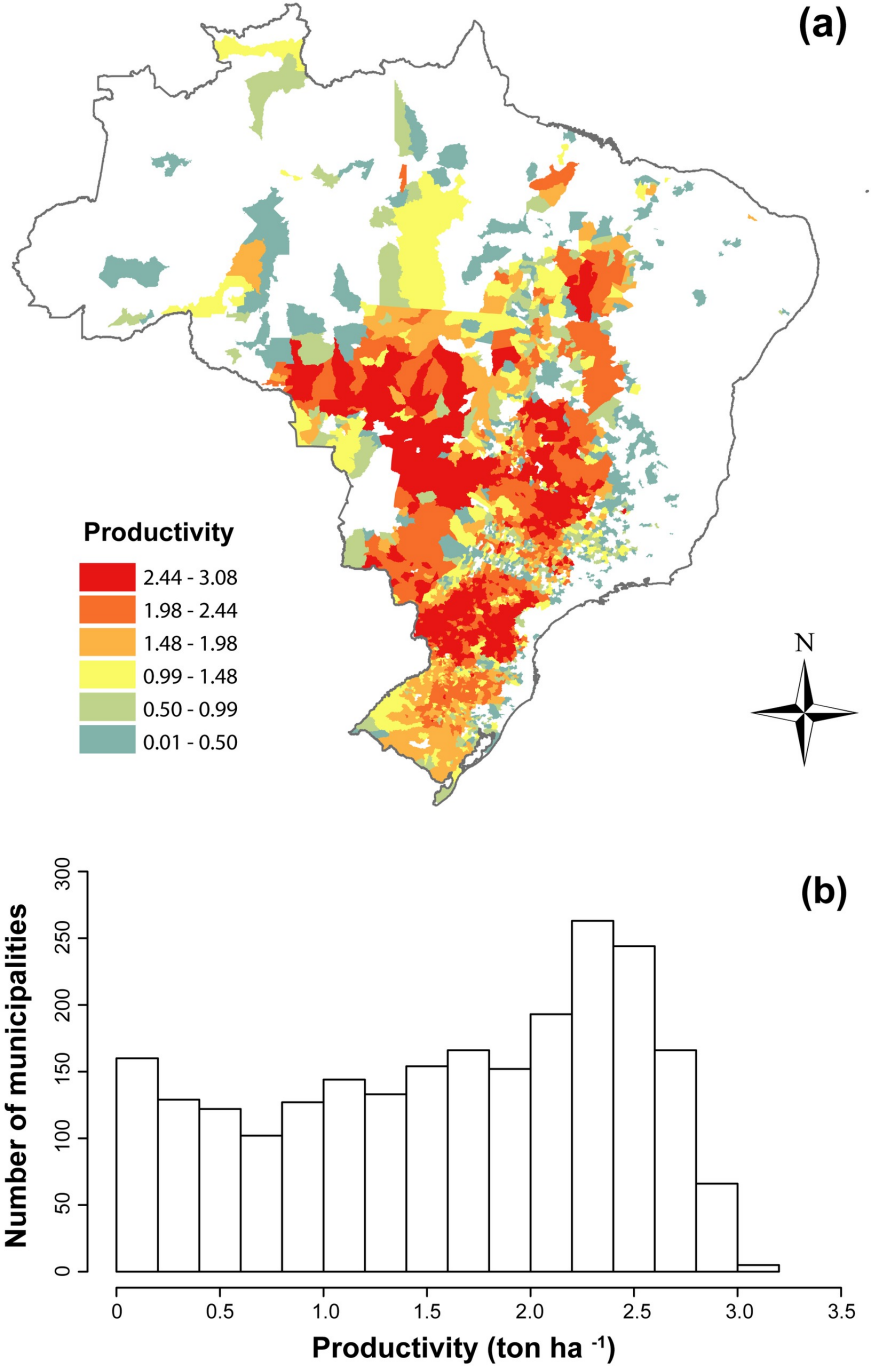
Soybean productivity in Brazilian municipalities. (A) Map of average soybean productivity in Brazilian municipalities from 1994 to 2010. (B) Distribution frequency of average soybean productivity (ton ha^-1^) by municipalities.

Soybean productivity for each Brazilian municipality was calculated by the ratio of the production (in tons) to harvested area (in hectares). Productivity values are given in tons per hectare (ton ha^-1^) for the period of 1994–2010. Production and harvested area values were obtained using the IBGE Automatic Recovery System (“Sistema IBGE de Recuperação Automática”—SIDRA; IBGE 2016 [57]) data bank. We identified a total of 2,304 Brazilian municipalities producers of soybean in this period. Average soybean productivity values (i.e., 1994–2010) are higher in the mid-southern region of Brazil (Fig 1A). The average soybean productivity distribution, as compared to the number of soybean producing municipalities (Fig 1B), has an asymmetric distribution, with few municipalities having the highest production. The productivity of 597 municipalities (~25% of total) ranged from 2.01 to 2.50 ton ha^-1^. Seven municipalities located in the southern region have the highest average productivity values (3.01 to 3.50 ton ha^-1^). Additionally, 174 of the 329 municipalities with average productivity, ranging from 2.51 to 3.00 ton ha^-1^, are found in Paraná (see S1 Table for productivity by municipalities).

The geographic coordinates (i.e., longitude and latitude) of the central town of each one of the Brazilian and other Latin American municipalities with average soybean productivity greater than zero (S1 Table) were used in ENMs. Thus, a total of 2,493 occurrence records distributed in Argentina, Paraguay, Bolivia, Ecuador, Venezuela and Brazil were obtained (see Figure A in S1 File). These records were overlaid on a spatial grid with 2,842 cells with a resolution of 0.5° x 0.5° covering the Neotropical region (see below). The other spatial analysis (eg., GWR, see below) were restricted to Brazil because they require records of soybean production for the Brazilian municipalities.

The efforts like genetic improvement and choice of cultivars, among other factors, can increase productivity and reduce climatic effects on productivity. From Brazilian soybean data, it is not possible to determine which variety was planted in each municipality. Additionally, the productivity data are temporal, but the cultivars may have changed over the years. In present paper we used occurrence records of soybean distributed along the Latin America to better describe the overall ecological niche of the species by ENMs (see below), therefore we contemplated all varieties used in each municipalities. Theses different varieties and their adjustments of local climatic conditions were used in ENMs. Therefore, the ENMs generated can be used to understand the current Soybean’s productivity of municipalities, however, for future climate scenarios, the ENMs should be evaluated with caution, since new varieties may emerge.

### Agricultural technology data for each municipality

Technological attributes can influence agricultural productivity, therefore, information on the technological efforts of each municipality can be used as surrogates of technological investment (equipment, specialized cultivars, among others) that would affect soybean productivity.

We selected 11 variables that indicate agricultural technological advances to be used in our models: 1) Individuals working in agricultural establishments; 2) Number of agricultural machinery equipment and implements existing in family agriculture establishments; 3) Number of agricultural machinery equipment and implements existing in non-family agricultural establishments; 4) Number of agricultural establishments that use electric energy; 5) Area of agricultural establishments (hectares); 6) Number of agricultural establishments with pest control and/or plant diseases; 7) Number of agricultural establishments with water resources; 8) Number of agricultural establishments using fertilizers; 9) Number of agricultural establishments using agrochemicals; 10) Area of agricultural establishments with irrigation use (hectares); 11) Number of agricultural establishments with irrigation use. The data were obtained from IBGE for the year 2006 (most updated census in Brazil).

### Ecological niche models

For modelling soybean suitability, we used climatic data scaled for the same grid used for the records. We obtained the bioclimatic variables for the pre-industrial time period by using simulations for the middle of the eighteenth century stabilized across a 200-year time period to represent current climatic conditions. Future climatic conditions for 2080–2100, a 20-year average for the end of the century, were based on the RCP4.5 emission scenarios, which were derived from four coupled Atmosphere-Ocean General Circulation Models (AOGCM). These were the Community Climate System Model (CCSM4), Centre National de Recherches Météorologiques (CNMR), Marine-Earth Science and Technology-National Institute for Environmental Studies (MIROC-ESM) and the Meteorological Research Institute (MRI-CGCM3). Climate data used in ENMs were obtained from EcoClimate database (ecoclimate.org [58]).

To generate ENMs, we selected five bioclimatic variables from 19 available variables (see Hijmans et al. [59]). This was based on a factor analysis that considered the correlation among the variables in a manner that minimized their collinearity (see Terribile et al. [60]). The climatic variables used in the ENMs were average annual temperature, extent of annual temperature, precipitation in the wettest month, precipitation in the driest month and precipitation in the warmest quarter. Additionally, we used soil pH for the depth of 30–100 cm (data obtained from the Harmonized World Soil Database–version 1.1, FAO/ IIASA/ISRIC/ISS-CAS/JRC 2009). Soil acidity is one of the major constraints of soybean production in Brazilian soils, as the acidity of these soils can affect plant growth and development by interfering with the availability of many nutrients required by plants, such as nitrogen, phosphorus and potassium [61]. In addition, it has been previously demonstrated that adding the pH variable improves ENMs for plant species [60,62,63]. Soil pH was treated as a continuous variable, used as a constraint variable and was kept constant over the time periods. The mean and amplitude values of the variables used can be found in Table A in S1 File.

We used ensemble methodologies [64] to determine the environmental suitability for soybeans in the present time period and to project it to the future. We followed the ensemble protocol proposed by Diniz-Filho et al. [65] and used by several recent papers dealing with ensembled ENM [63, 66, 67, 68, 69]. Twelve different ENMs were used, including six presence-only methods (i.e., BIOCLIM, Euclidian, Gower, Mahalanobis distances, Genetic Algorithm for Rule Set Production—GARP, and Maximum Entropy—MAXENT) and six presence-absence methods (i.e., Generalized Linear Models—GLM, Random Forest, Generalized Additive Models—GAM, Flexible Discriminant Analysis—FDA, Ecological Niche Factor Analysis—ENFA, and Neural Network). Franklin [70] and Peterson [35] provide general descriptions of the methods.

For model comparison, in both types of ENMs, i.e., presence-only and presence-absence, we used the same pseudo-absence data, but in the presence-only ENMs, pseudo-absences were used as background [69, 71]. We randomly divided the presence of each species and their pseudo-absences, which were randomly selected for a background region with the same proportion of species records (i.e., with a prevalence of 0.5), into 75% for calibration and 25% for evaluation and repeated this process 50 times. Based on thresholds determined by the ROC curve, the 2,400 resulting models (i.e., 50 cross-validation x 12 ENMs x four AOGCMs) were used to generate consensual occurrence maps. The ROC curve was based on the soybean frequency of occurrence in each neotropical grid cell, which was obtained from each ENM for every AOGCM (i.e., resulting in 48 frequency maps, from 12 ENMs x four AOGCMs) (for methodological details, see [63, 67, 69]. These 48 frequency maps were ensembled by their average and weighted by the True Skill Statistic (TSS, [72]; Table B in S1 File) into a single frequency map. This map was used as a measure of environmental suitability for soybeans across the neotropical region, ranging from 0, with no environmental suitability (i.e., the cell has no occurrence in any of the 48 models from the 50 randomizations) to 1, with maximum environmental suitability (i.e., the cell occurred in all models). The analyses were performed in the computational platform BioEnsembles [63, 65, 66, 67, 69, 71].

To inspect the main changes in suitability from the current to the future climate, we calculate the differences between the current and future suitability. Negative values indicate that municipalities will lose environmental suitability for soybean cultivation in the future scenario, whereas positive values denote the opposite. Zero values suggest that municipalities will not change their climate suitability values from the current scenario relative to the future.

In addition, we assessed the environmental similarity between calibration data and future climatic scenarios using extrapolation detection (EXDet) software [73]. The analysis detects two types of non-analogue conditions: type 1- points that fall outside the range covariates and type 2- points that are within the univariate range of climatic conditions but that constitute novel combinations of covariates [73]. This analysis identifies areas where models have extrapolated when making predictions and can help to more carefully interpret SDM results [74].

### Assessing the relative effect of environmental suitability and technology on soybean productivity

Soybean productivity, environmental suitability and all eleven technological variables were Z-transformed to facilitate the calculation of standardized local regression coefficients in a geographically weighted regression (GWR). Moreover, we used forward-selection for selected technological variables, so 7 out of the 11 initial variables were used (the variables 1, 2, 3, 4, 9, 10 and 11, described above).

We used GWR to investigate the relative influence of climate (suitability of ENM) and agricultural technology (seven variables) in explaining the spatial variation of soybean productivity. The GWR generates a regression model for each municipality, by using the entire dataset, and weighting the regression model by a geographic function, based on the spatial proximity of all other municipalities (see more details in Fotheringham et al. [47]). Thus, for each municipality, it was possible to determine all regression parameters (intercept, slope and R^2^). We performed three types of GWR: i) Using all predictor variables (GWRall); ii) Using only climate variables (GWRsuit); iii) Using only technological variables (GWRtec). In this way, we used the variation partitioning technique (see Legendre & Legendre 2012 [75]) based on the adjusted R^2^ to determine the relative influence of each predictor. The difference between GWRall and GWRtec indicates the pure effect of the climate variables, and the difference between GWRall and GWRsuit indicates the pure effect of the technology variables. The shared effect was determined as the difference between GWRtec and the pure effect of Technology (or GWRsuit and the pure effect of climate). The adjusted R^2^ of each pure effect and shared variables could thus be mapped and used to investigate how the relationship between the variables change across geographic space. To fit the GWR, we used a Bisquare spatial weight function with a fixed bandwidth of 5°. We quantified the improvement of the GWR with respect to an Ordinary Least Square (OLS) model using the adjusted Akaike Information Criterion (AICc). The GWR analyses were performed using Spatial Analysis in Macroecology (SAM), freely available at http://www.ecoevo.ufg.br/sam [76]. All shapefiles used in our figures are freely available at Ministério do Meio Ambiente (http://mapas.mma.gov.br/i3geo/datadownload.htm).

## Results

### Ecological niche modelling

**Fig 2:**
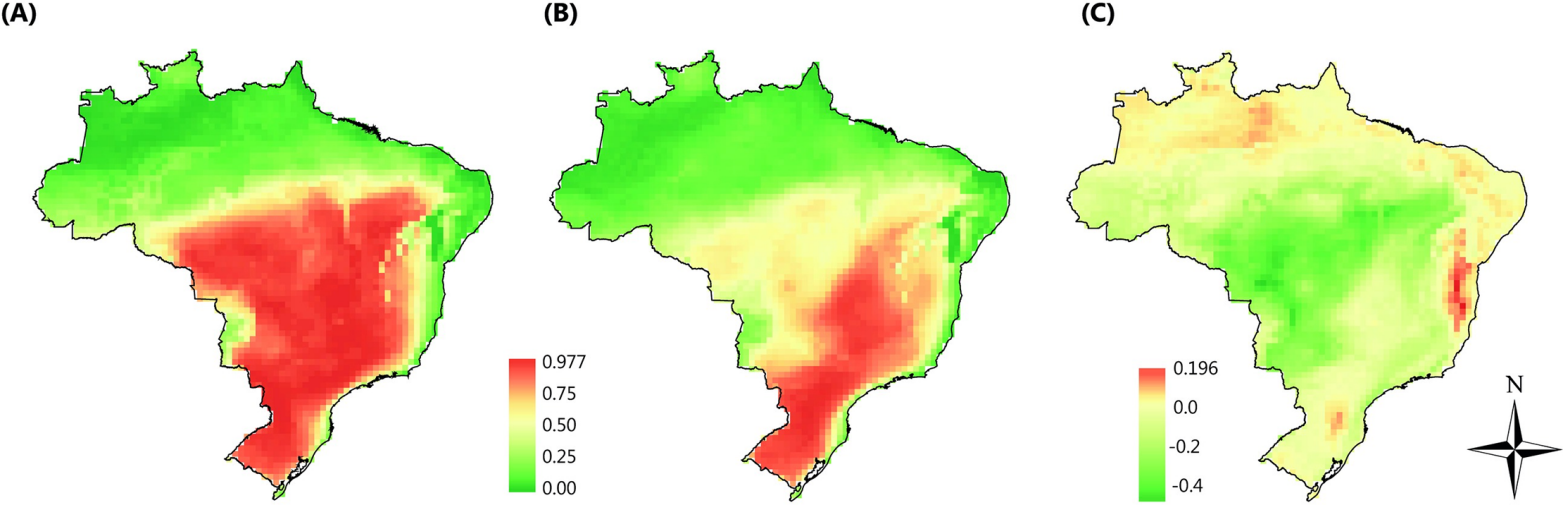
Average climate suitability of the ecological niche models for (a) current climate and (b) future climate scenarios, and (c) environmental suitability shifts of the future scenario when compared to the current scenario. Positive values in (c) indicates municipalities that will increase the environmental suitability for soybean cultivation in future scenarios.

In the current scenario, the environmental suitability for soybean cultivation (Fig 2A) reached its highest values in the central and southeast regions of Brazil, with values above 0.9 being considered favourable for soybean cultivation. On the other hand, northern and northeastern Brazilian municipalities had the lowest environmental suitability values, ranging from 0.0 to 0.2.

Climatically suitable areas in the future scenario (Fig 2) tend to reach their highest values in the south and southeast of Brazil, with values near 0.98. Actually, relative to the current scenario, over 72% of the Brazilian territory may lose environmental suitability for soybean cultivation in the future climate change scenario (Fig 2).

All future scenarios showed non-analogue climates to the current climate data of soybean areas (Figure B in S1 File). The type 1 novelty (at least one variable falling outside the training range) occurred in all scenarios. The MIROC climate model had the most regions with non-analogue climates. Type 2 novelty (new combinations of variables values) was rare, occurring in only 3 cells of the GISS and MIROC models.

### Geographically weighted regression

**Fig 3:**
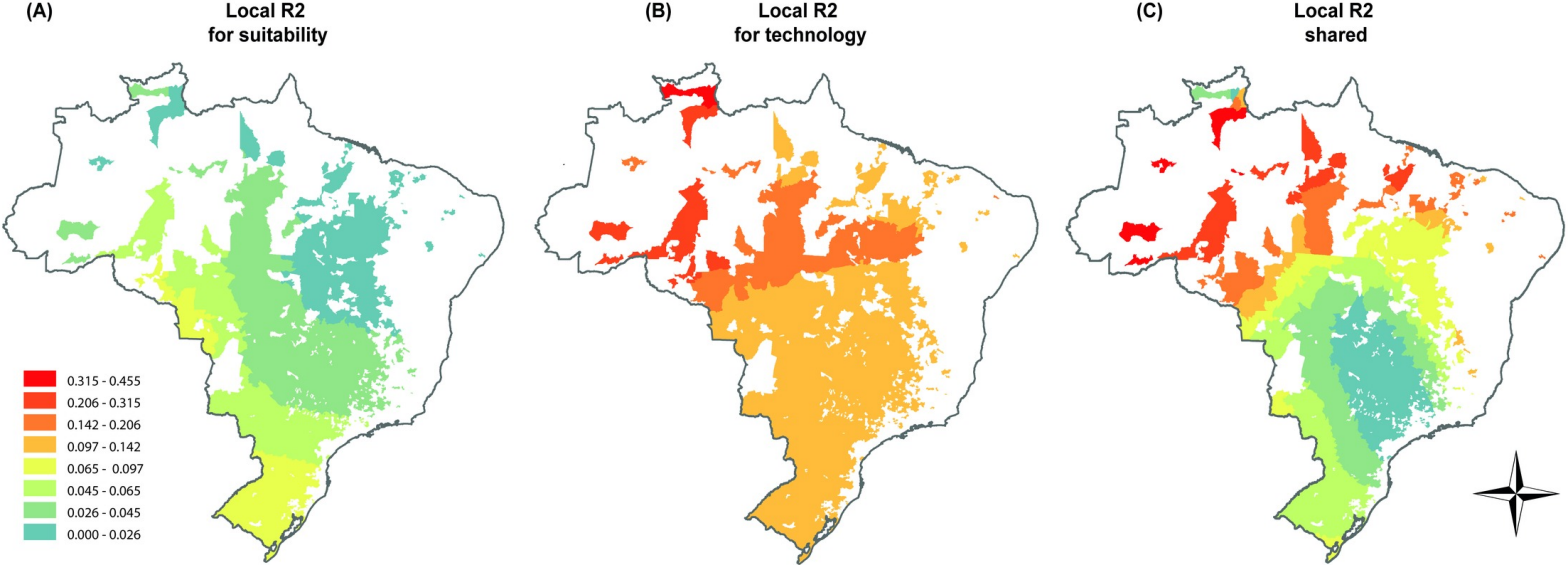
The partitioning of local adjusted R^2^ to explain soybean productivity in Brazilian municipalities. (A) Explained purely effect by Suitability; (B) Explained purely effect by Technology; (C) shared variation between Suitability and Tecnhology.

The actual environmental suitability and technology explained 30% of soybean productivity (GWRs, adjusted R^2^ = 0.3). Moreover, when comparing the GWR and OLS models (adjusted R^2^ OLS = 0.2), the AICc results showed that the GWR local model was significantly more appropriate than the OLS global model (F = 28.6 and P < 0.001, for the improvement of GWR over OLS). Using variation partitioning of the adjusted R^2^ obtained by GWR, we found that pure environmental suitability explained 5% (R^2^ = 0.05 P<0.05), and pure agricultural technology explained 11% (adjusted R^2^ = 0.11; P<0,05) of soybean productivity. The shared effect was 14% (adjusted R^2^ = 0.14). Moreover, partitioning for each municipality showed that the largest R^2^ occurred in a municipality in the southern and northern region of Brazil, with values widespread between the overall range of 0 to 0.1 (Fig 3A) On the other hand, the pure technology effect ranged from 0.1 to 0.44, with most of the highest values occurring in the north of the country (Fig 3B). The shared effect (climate and technology) showed the highest values in northern Brazil (Fig 3C). Thus, the GWR demonstrated that the influence of climate and agricultural technology on soybean productivity varies throughout Brazil. Nonetheless, technology was shown to be the most important factor to explain productivity values. Moreover, the regions with low environmental suitability (northern regions) showed the highest importance of agricultural technology.

## Discussion

Here, we showed that global climate change will impact the geographic distribution of soybeans in Brazil, mainly in the central and northern regions. Moreover, the productivity of municipalities was affected by climate and agricultural technology, though in different proportions for each predictor.

The use of ENMs to predict productivity using a linear model has been commonly used in many previous analyses (see Nabout et al. [40], for example, using only climate variables). However, ENMs can be biased because they assume a stationary spatial process. In other words, the relationship between predictors and species presence remains constant across the entire area under study. However, this is usually not the case, and non-stationary patterns are commonly found in geographical analyses [77]. Thus, techniques like GWR can be more useful than common regression to better evaluate the geographical variation of productivity. Moreover, the variation partitioning of the R^2^ of GWR can show the unique and shared effect of each predictor across the geographic space. In our analyses, climate was more important in explaining the productivity of soybeans in southern Brazil. This was where soybean cultivation was introduced to Brazil [52, 53], and thus, it is expected that this region would be more affected by climate variations. Nevertheless, this geographic area of Brazil (southern) has strong technological advances, which helps explaining the high soybean productivity in this region. This fact actually tends to support the overall use of niche models to predict the future distribution and cultivation of soybeans, coupled with information about the advances in technology applied to agriculture.

The investment in agricultural technology has been important in increasing the productivity of agriculture by reducing the negative effects of diseases, pests and climate change [78]. Moreover, the risks of climate change have intensified the importance of technology in agriculture [79]. In the present paper, technology was the main variable explaining soybean productivity in Brazil, mainly in northern Brazil. The introduction of soybeans in this region is more recent [52, 53], and technology has allowed productivity in a region with low environmental suitability. Despite the technological advances, climate change can lead to large socioeconomic changes (e.g. [80]), mainly because the technological resources demand financial expenses and the small farmer can have financial difficulty to have access to the technological resources [81].

In the present paper, the ecological niche models indicated vulnerable areas as well as new areas favourable to the cultivation of soybeans in Brazil. However, considering the impacts of climate change on ecosystems and biodiversity, we do not recommend new areas for the cultivation of soybeans. The conflict between the needs of food production and biodiversity conservation is normally more impactful on biodiversity and increases conflict around conservation (see [82]). For example, Bradley et al. [83] found that 40% of the areas surrounding protected areas in South Africa can be affected by changes in crop suitability, increasing threats for conservation and generating social conflicts. Our results show that the higher values of future environmental suitability for soybeans in Brazil would be located in the Atlantic forest biome, considered a biodiversity hotspot [84]. It would be, at the very least, questionable to consider expanding soybean cultivation to these areas as an option due to the priority given to Atlantic forest conservation. In addition, the Atlantic forest is the most populous region of Brazil, sheltering approximately 72% of Brazilian population [85], which means that, in this area, less space at much higher economic cost would be available for cultivation.

A solution we advocate to overcome the impacts of climatic change on crop production is the adoption of technological strategies to adapt to climate impacts in a way that increases the production of actual cultivation areas or, at least, minimizes productivity losses before the negative impacts become too severe and expensive to be reversed [16]. Examples would be altering the sowing of the species in order to avoid water stress during the initial growing phases [86, 87], the development of new cultivars and hybrids tolerant to local abiotic stresses (e.g., droughts and extreme temperatures) [12, 88], intercropping with different crops [21], the use of cover crops to diminish soil warming, and changing the spacing and seed rate [14]. More recently, the use of Ecosystem-based Adaptation (EbA) practices can potentially assist farmers in reducing the impacts of climate change on agricultural production (see [89, 90]). EbA practices are based on conservation, management of biodiversity and ecosystem services in a way that reduces the effects of climate change and promotes social, economic and environmental improvement [91]. Studies about how and which climate variables will change in the future, and how soybean varieties are impacted by them, are necessary to define which of the abovementioned strategies will be more effective for soybean production in Brazil.

### Caveats for the use of ecological niche models

It is important to note that ENMs—as many other predictive models—are known to have uncertainties inserted in all stages of the modelling processes, that can, in turn, affect their results [68, 92]. For example, the input data can be climatic biased or inaccurate [93, 94]. For crop species, absence of cultivation or productivity in some regions can be due to socioeconomic aspects [95, 96], rather than the lack of climatic favorable conditions. Thus, the occurrences/productivity could be biased for some regions and consequently, for some climatic conditions, which could generate erroneous response curve of the relationship of species occurrence and the climatic variables. Here we consider that due to the economic importance of soybean its currently distribution represents the overall climatic conditions that permits its cultivation, thus that our samples are not biased related to soybean requirements. However, other factors unrelated with climatic variables not included in our models as interaction with other species, soil types, irrigation or other historical processes affects the distribution and productivity of cultivars and can also be a source of variation [10, 97]. Furthermore, ENMs predictions for future scenarios should be interpreted with caution, as climate change can lead to novel combinations of climatic variables, that are different from those used for model calibration, which associated with uncertainty about the future atmospheric CO_2_ and its fertilization effects on plants productivity, increase uncertainty of ENMs results [98, 99, 39]. Although it is important to be aware about these limitations when interpreting ENMs results, they should not prevent the use of ENMs, as even with such drawbacks when compared with more complex and detailed models ENMs had led to similar conclusions, using simpler and easy to get data [10].

## Conclusions

Agricultural technology and climate are important factors explaining soybean productivity in Brazilian municipalities. Moreover, some regions can be more affected by climate and, consequently, climate change. Although the environmental suitability of some areas would increase, there was an overall decrease in environmental suitability, indicating that soybean cultivation in Brazil could be highly threatened in the future. Considering the importance of Brazil soybean cultivation for the global supply, our results highlight the imminent need to develop strategies to mitigate the impacts of climate change and maintain high productivity in the actual cultivated areas. Thus, for areas that may loose productivity, we recommend programmes such as Ecosystem-based Adaptation (EbA; see [88]). For areas where suitability will increase, since the environmental conditions are more favourable, we suggest studies that focus on the development of strategies to increase productivity and reduce costs (social and environmental). We do not suggest the exploration of new areas in any of these cases, as this can increase environmental and food degradation and consequently affect overall human wellbeing.

## Supporting information

S1 Table Data about the municipalities used in present papers. (XLSX)

S1 File Information on methods and results of ecological niche model of soybean. (DOCX)
