# Supplementary material for: Geographical patterns in climate and agricultural technology drive soybean productivity in Brazil
Source: PLoS One. 2018 Jan 30;13(1):e0191273. doi: 10.1371/journal.pone.0191273 (PMC5790230; doi:10.1371/journal.pone.0191273)
Supplement: S1 File — (DOCX) [file pone.0191273.s002.docx]

**Supplementary Material**

**

**

**Figure A. Occurrence point used in Ecological Niche Model**

**Table A:** Mean, maximum, and minimum values of bioclimatic variable for each atmosphere-ocean general circulation model (AOGCM) with their respective time periods (time). Soil pH value does not have difference related with AOGCM and Time and was put separate.

| **AOGCM** | **Time** | **Bioclimatic variable** | **Mean** | **Maximum** | **Minimum** |
| --- | --- | --- | --- | --- | --- |
| **-** | - | Soil pH | 5.843723 | 8.92279 | 0 |
| CCSM | Present | Bio 1, annual mean temperature | 20.30104 | 28.55201 | 1.526303 |
| CCSM | Present | Bio 2, mean diurnal range | 8.412115 | 13.31255 | 0.531448 |
| CCSM | Present | Bio 3, isothermality | 55.3277 | 80.64586 | 12.30898 |
| CCSM | Present | Bio 16, precipitation of the wettest quarter | 648.6321 | 2354.237 | 56.64392 |
| CCSM | Present | Bio 17, precipitation of the driest quarter | 118.3395 | 769.512 | 2.553541 |
| CCSM | Future | Bio 1, annual mean temperature | 22.97953 | 31.26831 | 3.949623 |
| CCSM | Future | Bio 2, mean diurnal range | 8.78543 | 13.75214 | 0.530173 |
| CCSM | Future | Bio 3, isothermality | 55.20164 | 80.44295 | 12.05746 |
| CCSM | Future | Bio 16, precipitation of the wettest quarter | 651.7339 | 2474.877 | 48.32971 |
| CCSM | Future | Bio 17, precipitation of the driest quarter | 111.665 | 862.5688 | 1.129947 |
| GISS | Present | Bio 1, annual mean temperature | 22.69849 | 31.63193 | 2.673582 |
| GISS | Present | Bio 2, mean diurnal range | 7.857951 | 16.91281 | 1.057245 |
| GISS | Present | Bio 3, isothermality | 56.41578 | 86.12522 | 16.45541 |
| GISS | Present | Bio 16, precipitation of the wettest quarter | 569.2506 | 1382.33 | 48.06391 |
| GISS | Present | Bio 17, precipitation of the driest quarter | 176.3608 | 804.6979 | 6.16132 |
| GISS | Future | Bio 1, annual mean temperature | 24.59036 | 34.00086 | 4.236327 |
| GISS | Future | Bio 2, mean diurnal range | 7.85296 | 16.16819 | 1.04355 |
| GISS | Future | Bio 3, isothermality | 56.70525 | 87.34168 | 16.11781 |
| GISS | Future | Bio 16, precipitation of the wettest quarter | 553.432 | 1486.013 | 45.69493 |
| GISS | Future | Bio 17, precipitation of the driest quarter | 167.8489 | 876.8783 | 7.210345 |
| MIROC | Present | Bio 1, annual mean temperature | 21.15673 | 29.45503 | 5.113974 |
| MIROC | Present | Bio 2, mean diurnal range | 8.535584 | 18.0589 | 0.421653 |
| MIROC | Present | Bio 3, isothermality | 51.9316 | 78.47282 | 13.36875 |
| MIROC | Present | Bio 16, precipitation of the wettest quarter | 556.7297 | 1256.346 | 42.55663 |
| MIROC | Present | Bio 17, precipitation of the driest quarter | 85.26761 | 752.6424 | 1.939451 |
| MIROC | Future | Bio 1, annual mean temperature | 24.6314 | 32.2284 | 7.06493 |
| MIROC | Future | Bio 2, mean diurnal range | 9.167496 | 18.09284 | 0.427563 |
| MIROC | Future | Bio 3, isothermality | 53.12911 | 81.35944 | 12.80794 |
| MIROC | Future | Bio 16, precipitation of the wettest quarter | 540.1736 | 1138.598 | 26.77511 |
| MIROC | Future | Bio 17, precipitation of the driest quarter | 85.77835 | 758.1906 | 1.215983 |
| MRI | Present | Bio 1, annual mean temperature | 20.39737 | 27.54746 | 1.277362 |
| MRI | Present | Bio 2, mean diurnal range | 7.215411 | 12.22014 | 0.447526 |
| MRI | Present | Bio 3, isothermality | 57.11404 | 86.77493 | 14.11594 |
| MRI | Present | Bio 16, precipitation of the wettest quarter | 747.5988 | 1982.2 | 21.86692 |
| MRI | Present | Bio 17, precipitation of the driest quarter | 149.1608 | 871.1057 | 1.417165 |
| MRI | Future | Bio 1, annual mean temperature | 22.37575 | 30.54558 | 3.645514 |
| MRI | Future | Bio 2, mean diurnal range | 7.088678 | 13.15855 | 0.456378 |
| MRI | Future | Bio 3, isothermality | 54.66489 | 88.45825 | 12.29391 |
| MRI | Future | Bio 16, precipitation of the wettest quarter | 779.0453 | 2318.087 | 22.97626 |
| MRI | Future | Bio 17, precipitation of the driest quarter | 131.8256 | 808.2545 | 0.354293 |

**Table B**: Values of True Skill Statistics (TSS) of the combination between each ecological niche model (ENM) and atmosphere-ocean general circulation model (AOGCM). TSS values of the ensemble ENMs was also shown

| **ENM** | **AOGCM** | | | |
| --- | --- | --- | --- | --- |
|  | *CCSM* | *GISS* | *MIROC* | *MRI* |
| BioClim | 0.5394 | 0.4702 | 0.4835 | 0.3946 |
| EuclidDist | 0.4451 | 0.6171 | 0.4952 | 0.5327 |
| GowerDist | 0.3937 | 0.6089 | 0.4067 | 0.5013 |
| MahalanobisDist | 0.4051 | 0.5968 | 0.34 | 0.5124 |
| GLM | 0.6232 | 0.6641 | 0.5867 | 0.6038 |
| MaxEnt | 0.6619 | 0.6956 | 0.7086 | 0.6752 |
| RndFor | 0.6695 | 0.6771 | 0.6606 | 0.5854 |
| GAM | 0.6644 | 0.641 | 0.6492 | 0.6663 |
| FDA | 0.6286 | 0.6898 | 0.6044 | 0.6406 |
| MARS | 0.6159 | 0.654 | 0.6403 | 0.6384 |
| ENFA | 0.374 | 0.4346 | 0.4705 | 0.5968 |
| NNet | 0.5743 | 0.586 | 0.5679 | 0.6492 |
| Ensembled | 0.4311 | 0.4473 | 0.441 | 0.4425 |


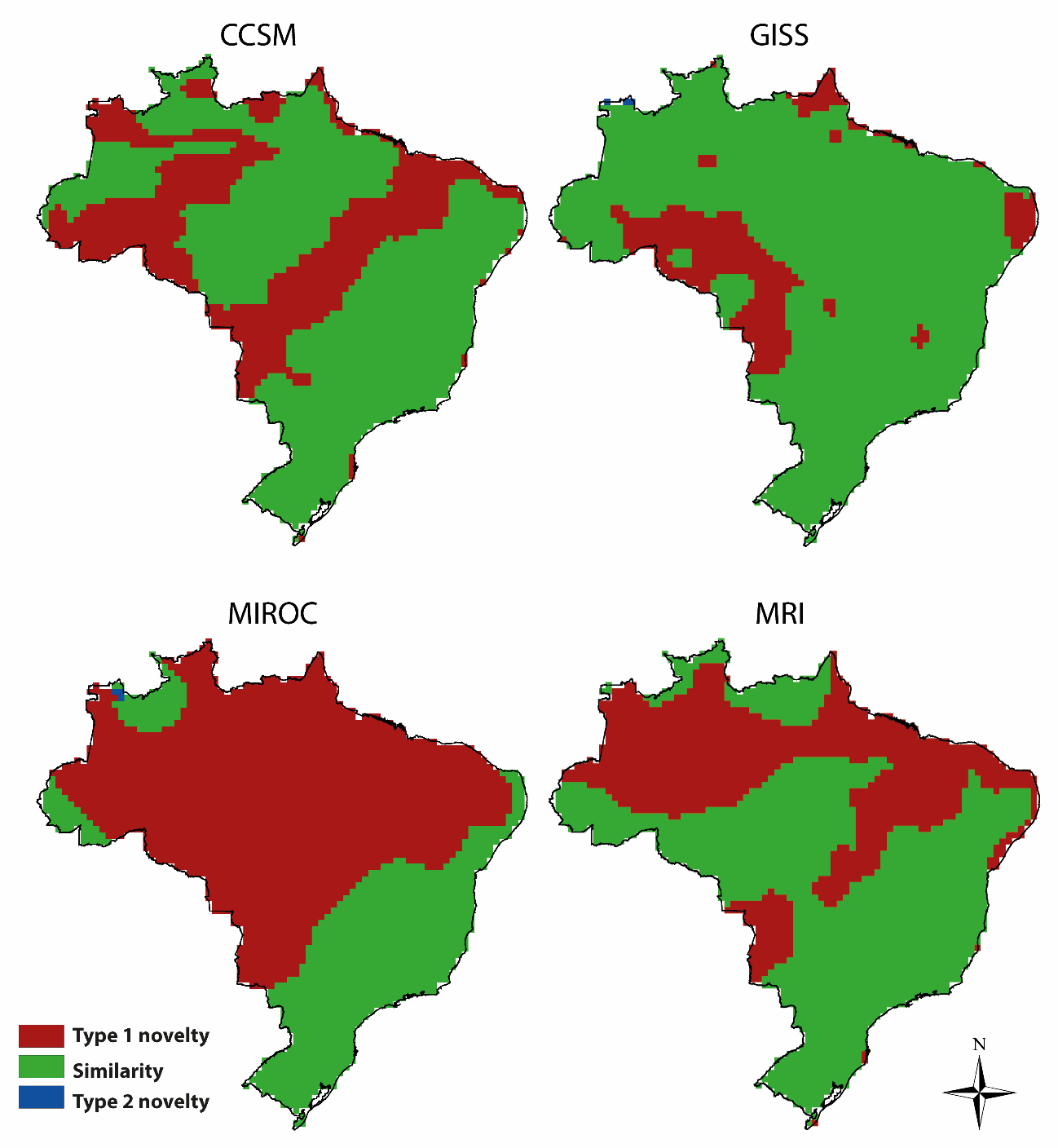


**Figure B**: Map showing the extrapolation projection areas for soybean. The type 1 novelty indicate areas where at least one climate variable is outside the range of training data. Type 2 novel indicate areas where climatic variables are within the range of training data, but that represent new combinations of variables values. Areas in which climatic data are in the same range and represent the same covariates combinations, i.e. areas that are similar to training data are represented in green.
